# An efficient method for measuring plasma volume using indocyanine green dye

**DOI:** 10.1016/j.mex.2019.05.003

**Published:** 2019-05-08

**Authors:** Sixtus Aguree, Alison D. Gernand

**Affiliations:** Department of Nutritional Sciences, The Pennsylvania State University, 110 Chandlee Laboratory, University Park, PA, 16802, United States

**Keywords:** Measuring plasma volume using ICG, Plasma volume, Blood volume, Indocyanine green, Nutritional biomarker, Nonpregnant women

## Abstract

Plasma volume (PV) can be an important marker of health status and may affect the interpretation of plasma biomarkers, but is rarely measured due to the complexity and time required. Indocyanine green (ICG) is a water-soluble tricarbocyanine dye with a circulatory half-life of 2–3 min, allowing for quick clearance and repeated use. It is used extensively in medical diagnostic tests including ophthalmologic imaging, liver function, and cardiac output, particularly in critical care. ICG has been validated for measuring PV in humans, however previous work has provided minimal published details or has focused on a single aspect of the method. We aimed to develop a detailed, optimal protocol for the use of ICG to measure PV in women of reproductive age. We combined best practices from other studies and optimized the protocol for efficiency.

• This method reduces the time from blood collection to PV determination to ˜2 h and the amount of plasma required to estimate PV to 2.5 mL (1.5 mL before ICG injection and 1.0 mL post-injection).
• Participant inconvenience is reduced by inserting an intravenous (IV) catheter in only one arm, not both arms.
• Five post-injection plasma samples (2–5 min after ICG bolus) are enough to accurately develop the decay curve for plasma ICG concentration and estimate PV by extrapolation.

## Graphical abstract

**Figure:**
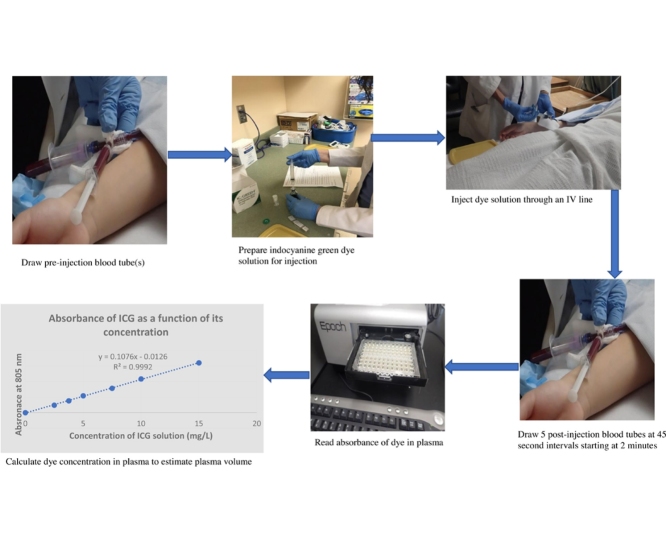

**Specifications Table**

**Table:** 

Subject Area:	*Medicine and Dentistry*
More specific subject area:	*Blood and plasma volume physiology*
Method name:	*Measuring plasma volume using ICG*
Name and reference of original method:	Bradley, E.C. and J.W. Barr, *Determination of blood volume using indocyanine green (cardio-green) dye.* Life Sciences, 1968. 7(17): p. 1001–7. Schad H, H.M., Brechtelsbauer H., *Determination of plasma volume with indocyanine green.* Der Anaesthesist, 1987. 36(11): p. 608–14. Haller, M., C. Akbulut, H. Brechtelsbauer, W. Fett, J. Briegel, U. Finsterer, et al., *Determination of plasma volume with indocyanine green in man.* Life sciences, 1993. 53(21): p. 1597–604. Jacob, M., P. Conzen, U. Finsterer, A. Krafft, B.F. Becker, and M. Rehm, *Technical and physiological background of plasma volume measurement with indocyanine green: A clarification of misunderstandings.* Journal of applied physiology (Bethesda, Md.: 1985), 2007. 102(3): p. 1235–42. Polidori, D. and C. Rowley, *Optimal back-extrapolation method for estimating plasma volume in humans using the indocyanine green dilution method.* Theoretical Biology and Medical Modelling, 2014. 11: p. 33.
Resource availability:	*If applicable, include links to resources necessary to reproduce the method (e.g. data, software, hardware, reagent)*

## Methods

### Introduction

Plasma is the water component of blood in which nutrients, hormones, and other biomarkers circulate. Plasma volume (PV) is the total amount of plasma in blood. It is an important biomarker in pregnancy, in chronic heart failure patients, as well as in situations where blood transfusion is critical. PV changes to a small extent from temperature changes and exercise [[1], [2], [3]], and some evidence has shown changes in PV at different points in the menstrual cycle [4,5]. In pregnancy, PV increases on average by 50% from non-pregnant values, causing hemodilution [6]. Abnormal PV expansion has been associated with adverse pregnancy outcomes [[7], [8], [9], [10]]. Because proteins and biomarkers of health are transported in the plasma, the amount of PV could impact how biomarker concentrations are interpreted.

Despite the substantial value of PV assessment, it is not routinely measured in clinical settings or during pregnancy, partly because the method is cumbersome and time consuming. PV could be a useful diagnostic tool if it could be safely and easily measured. The method recommended by the International Committee for Standardization in Hematology (ICSH) for measuring PV uses radioiodine-labeled human serum albumin (^125^I–HSA) [11]. ^131^I–HSA has also been used for PV measurement [12]. It is not ethical to use radioactive tracers in some populations including children and pregnant women. As well, it is challenging and expensive to perform measurements with such tracers. Other methods using dyes that bind to albumin, and therefore distribute throughout the vascular space with albumin, have been developed. The two main dyes used are Evans blue [13] and indocyanine green (ICG) [14], each of which have been validated against ^125^I–HSA in humans. Evans blue dye is no longer available for purchase in the US [15]. ICG is currently produced and sold and is advantageous because it is rapidly cleared from circulation. ICG also has a short circulatory half-life of 2.5–3 min [16,17]. These properties allow for quick assessment and repeated measurements of plasma ICG concentration and PV within a day [18], even as early as 30 min following the first injection [16,19]. In clinical settings, ICG has been used extensively in ophthalmologic imaging to examine the eye structure [[20], [21], [22]].

Most PV measurements specify that the dye (tracer) be injected in one arm and blood collected on the contralateral arm to avoid possible contamination of the tracer with post-injection blood [16]. This has been followed by others [23]. The inconvenience and challenge of inserting an intravenous (IV) catheter line in both arms can be overcome by replacing all or parts of the blood collection system following the dye injection, before post dye-injection blood is collected [24]. The objective of this study was to develop an efficient and less-invasive ICG method for measuring PV in women of reproductive age by combining best practices from the literature and further reducing 1) the number of blood samples needed and 2) the amount of plasma needed per subject.

### Study design

We conducted a single visit study at Penn State to develop and test an ICG method for measuring PV in non-pregnant women of reproductive age. Currently, PV measurement is not one of the Food and Drug Administration (FDA) approved uses of ICG but the method uses ICG in the same manner as FDA approved uses such as cardiac output, liver function and hepatic blood flow. As a result, our work was granted an investigational new drug (IND) exemption from the FDA. ICG has been used safely in pregnancy, and does not appear to cross the placenta [25,26] but is not currently approved for use in pregnant women in the US. Thus, our protocol in women of reproductive age includes a pregnancy test the same day of the measurement (we additionally screened out women who were trying to get pregnant).

Visits were conducted in the Clinical Research Center (CRC), a service unit in The Pennsylvania State University’s Clinical and Translational Science Institute, University Park, PA. Participants were healthy women of reproductive age that were non-pregnant, non-breastfeeding, and not using hormonal birth control methods. Eligibility also included normal blood pressure (systolic blood pressure 90 to <140 mmHg and/or diastolic blood pressure 60 to <90 mmHg) because we wanted healthy subjects, and because there are previous reports of blood pressure dropping after ICG use [27,28]. The study visit was scheduled to occur within the early follicular phase of the menstrual cycle, aiming for cycle day 2 (because previous studies have found variation in PV across the menstrual cycle) [4,5]. Two days before the study visit, participants were asked to drink plenty of water and to abstain from any form of alcohol because we wanted each person to be well hydrated at the time of measurement. All participants fasted for 12 h (overnight) before the study visit, which took place in the morning between 7 a.m. and 10 a.m. This component of the protocol was needed to standardize methods for biomarker measurement (not described here), but it also served to standardize the timing of PV measurement.

At the visit, women were asked a series of questions about their health history and lifestyle. Blood pressure, height, and weight were measured using standard methods. Weight was needed to calculate the amount of ICG solution to inject. Participants provided a fresh urine sample for a human chorionic gonadotropin (hCG)-based pregnancy test. We measured body fat percentage using the Tanita InnerScan Body Composition Monitor.

After these measurements, the specific protocol for ICG measurement of PV began.

### Blood collection and processing

Women rested for 15 min in a supine position in a hospital-style room with a small heating pad over the inside of the arm selected for IV insertion (either arm was used, based on participant preference). At the end of 15 min, a temporary tourniquet was applied to aid in identifying an antecubital vein and an IV (BD Insyte Autoguard 18 G and 20 G, size of gauge varied depending on size of the participant, Becton, Dickinson and Company, Franklin Lakes, NJ, USA) was inserted by a nurse. To standardize the point of entry, we only used the antecubital vein and did not consider other locations (e.g., hand or wrist) if the IV was unsuccessful in the arm. After IV insertion, blood was drawn into a 6 mL vacutainer blood collection tube coated with K_2_ ethylenediaminetetraacetic acid (K_2_ EDTA). Of note, a 4 mL tube would be sufficient for measuring PV, but we took extra plasma to aid in method development. We also collected blood into tubes for serum and whole blood at this time for measurement of other biomarkers. After ICG injection, 5 more EDTA tubes were collected as described below. We used 3 mL tubes for method development, but 2 mL would be more than enough for our final method. EDTA tubes were gently inverted 10 times immediately after the tube was filled. Tubes were centrifuged (within 10 min of collection) at 3200 rpm for 15 min (Quest Diagnostics, Horizon model 642E centrifuge) to separate plasma from blood cells. The plasma samples were transported to the laboratory to determine the ICG concentrations. The PV was calculated from the laboratory-measured ICG values. PV determination was completed within 2 h of blood collection.

### Indocyanine green injection and post-injection blood collection

ICG doses up to 5.0 mg/kg body weight have been reported to be safe in humans [29]. This amount has been used in pregnant women without any adverse effects [25]. Haneda et al. used between 5.0 to 10.0 mg in children and 10.0 to 15.0 mg ICG in adults [13]. Other researchers have used 25.0 mg to study ICG clearance by the liver [30]. The most commonly used doses for injection in the determination of PV and ICG plasma disappearance rate studies are 0.25 mg/kg body weight [14,23,31,32] or 0.50 mg/kg body weight [30,[33], [34], [35], [36], [37]]. Plasma disappearance rates of ICG using 0.25 mg/kg body weight are comparable to 0.50 mg/kg body weight [38]. In this study, we chose the lower dose of 0.25 mg/kg because it is expected that lower doses will clear faster from the body than higher ones, and it will use less ICG overall.

For this study, we used an ICG kit that contained a 10 mL ampule of sterile water and 25 mg of ICG powder in a vial (IC-Green®, AKORN Inc, Lake Forest, IL, USA). The study nurse added the water to the ICG vial to create a concentration of 2.5 mg/mL, immediately before injection. The solution must be used within 6 h of mixing, so we waited until the IV was successfully placed for each participant before mixing the ICG and water. A 10 mL syringe (Luer-Lok™) was rinsed with the ICG solution (˜1 mL) and the calculated volume (0.25 mg/kg x body weight (kg) ÷ 2.5 mg/mL) was drawn for injection. The 10 mL syringe was weighed with the full content for injection, then reweighed after the injection to determine the exact weight of the ICG injected using a high-precision scale. The weight of the ICG injection was used later for PV determination (see below).

**Fig. 1:**
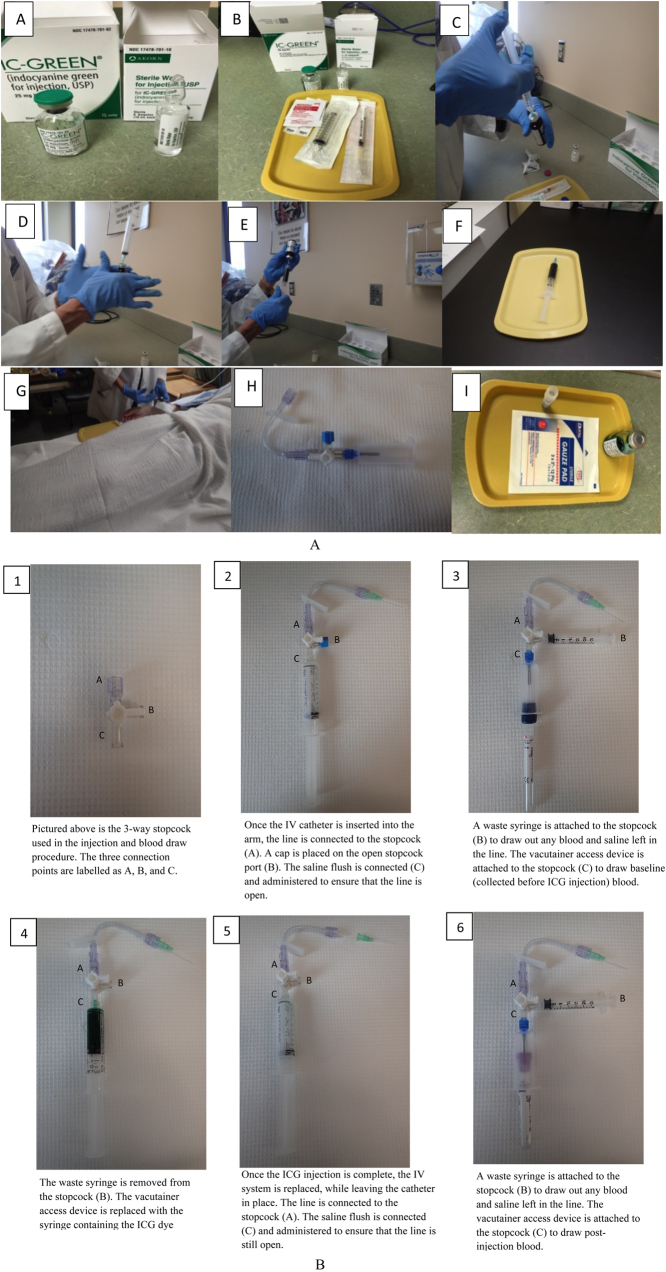
(a) ICG preparation and injection: A, ICG powder and sterile water; B, syringes and alcohol pad for disinfecting; C, transferring the sterile water into the ICG powder vial to prepare the ICG solution; D, mixing the ICG power and sterile water to ensure that all the power is dissolved; E, pipetting the required volume of ICG solution for injection; F, ICG solution ready for injection; G, injecting ICG solution into the IV line established; H, 3-way stopcock replacement system for blood collection; I, Remaining ICG solution in vial and the aliquoted ICG solution (in cryovial) for lab work. (b) Steps for 3-way stopcock connections during the ICG injection and blood collection process.

A bolus dose of ICG (0.25 mg/kg body weight) was injected evenly over 5 s into the antecubital vein through an IV line with a 3-way stopcock (Baxter Healthcare Corporation, Deerfield, IL, USA) attached, and flushed with 10 mL saline (McKesson Medical-Surgical, Inc., Richmond, VA, USA). The 3-way stopcock (with rotating male luer lock) was replaced after the dye injection to prevent contamination of ICG and post-injection blood to be collected (see Fig. 1a and b and Suppl. 1). A timer was started at the beginning of ICG injection (and used to count out loud 5 s as the nurse performed the injection). Starting at 2 min, blood samples were collected into 3 mL EDTA vacutainer blood collection tubes every 45 s, up to 5 min (total of 5 draws at exactly (min:sec) 2:00; 2:45; 3:30; 4:15; and 5:00). The time in seconds was recorded at each draw. The method is still successful if the draw is not evenly spaced at each interval (but it is very important to know the exact time of each draw). Blood was drawn into 2 mL syringes (connected to the 3-way stopcock) before each draw and pushed back immediately after the draw to keep the IV line clear (Suppl. 1 video). The blood collection tubes and syringes were purchased from BD (Becton, Dickinson and Company, Franklin Lakes, NJ, USA). Blood samples were processed the same way as the 6 mL EDTA tubes described above, and were used for PV determination described below.

This is an overview of the full process:

#### Participant visit

Collect urine sample for pregnancy test; weigh participant; take blood pressure.

Have participant rest in a supine position for 15 min, with a small heating pad over the antecubital vein (for temperature control and to aid IV insertion).

Trained phlebotomist inserts an intravenous (IV) catheter with a 3-way stopcock (Baxter Healthcare Corporation, Deerfield, IL, USA).

Participant rests supine for 5 min. [During this time, ICG powder and water are mixed, and appropriate volume per participant’s weight (at 0.25 mg/kg) is drawn into a syringe. Keep remaining ICG mixture for lab work.]

Collect pre-injection blood sample(s) – minimum 4 mL blood in EDTA vacutainer tube needed for plasma. Collect additional blood here if other biomarkers will be measured.

Start timer and inject ICG through IV in a bolus dose over 5 s.

Flush with 10 mL saline solution.

Remove and replace 3-way valve system (3-way stopcock).

Attach a 2 mL syringe to the 3-way stopcock. Before each blood draw, draw blood into the syringe and replace this after each tube is drawn.

At exactly 2 min from start of ICG-injection, draw 2 mL blood using EDTA vacutainer tube; continue with 4 more blood draws every 45 s (will end at exactly 5 min post injection). Use a larger blood tube here (e.g., 3 mL) if other biomarkers will be measured.

Remove IV and let participant get up when comfortable; take blood pressure. [Participant involvement is complete]

#### Laboratory procedure

Process blood tubes per standard centrifugation methods; aliquot plasma.

Set up the standard curve and 96-well plate per details below.

Measure ICG wavelength (805 nm) in a standard plate reader.

Plot the standard curve and the decay curve for plasma ICG concentration.

Extrapolate the decay curve for plasma ICG concentration to estimate plasma volume.

### PV determination

PV was measured by applying the indicator-dilution principle. Plasma obtained from the participant before injection (“blank”) and the five plasma samples obtained after the injection of ICG were used to determine PV for each subject. Calibration curves [39] were prepared by diluting the initial concentration of ICG (2.5 mg/mL) with MilliQ water (EMD Millipore Corporation, Billerica, MA, USA) to concentrations of 5 mg/L–30 mg/L (Table 1). A solution with 200 μL of ICG and 200 μL of the participant’s blank plasma were mixed together to obtain final standard concentrations of 2.5 mg/L–15 mg/L (Table 2). We chose this range to so that absorbance readings from different subjects and clearance could be captured. The linear relationship between absorbance and concentration of ICG solution in plasma follows the Beer-Lambert’s law up 15 mg/L [40]. All samples including standard solutions, blank samples and post-injection plasma samples were vortexed at high speed for 10 s for even mixing of solutes, and 100 μL of each was pipetted into 96 well plates, in triplicate. Absorbance was read on an Epoch plate reader (BioTek Instruments, Inc., Winooski, VT, USA) powered by Gen5™ Software, set to a wavelength of 805 nm. Triplicate readings were taken for each blank, standard, and sample. The mean of 3 readings was calculated and used as the result.

**Table 1:** Preparation of standard concentrations for ICG (using six 25 mL volumetric flasks, total volume for each is 25 mL).

Standard concentrations (mg/L)	Volume of 2.5 mg/mL ICG solution to add (μL)	Volume of milliQ water (μL)	
5	50	24,950	Mix thoroughly
7.5	75	24,925	
10	100	24,900	
15	150	24,850	
20	200	24,800	
30	300	24,700

**Table 2:** Final concentration for standard curve (using six 1.5 mL microcentrifuge tubes, total volume for each is 400 μL).

Standard concentrations (mg/L) (from Table 1)	Amount of standard (μL) (from Table 1)	Amount of pre-injection plasma to add (μL)		Final concentration of standards (mg/L)
5	200	200	Vortex at high speed for 10 s	2.5
7.5	200	200		3.75
10	200	200		5
15	200	200		7.5
20	200	200		10
30	200	200		15

**Fig. 2:**
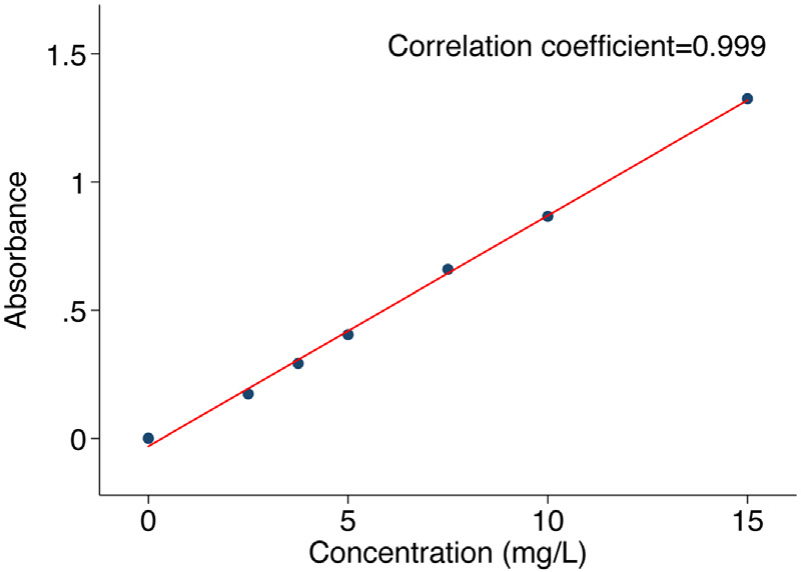
Standard curve for estimating the concentration of ICG in unknown plasma samples (example from one participant).

**Fig. 3:**
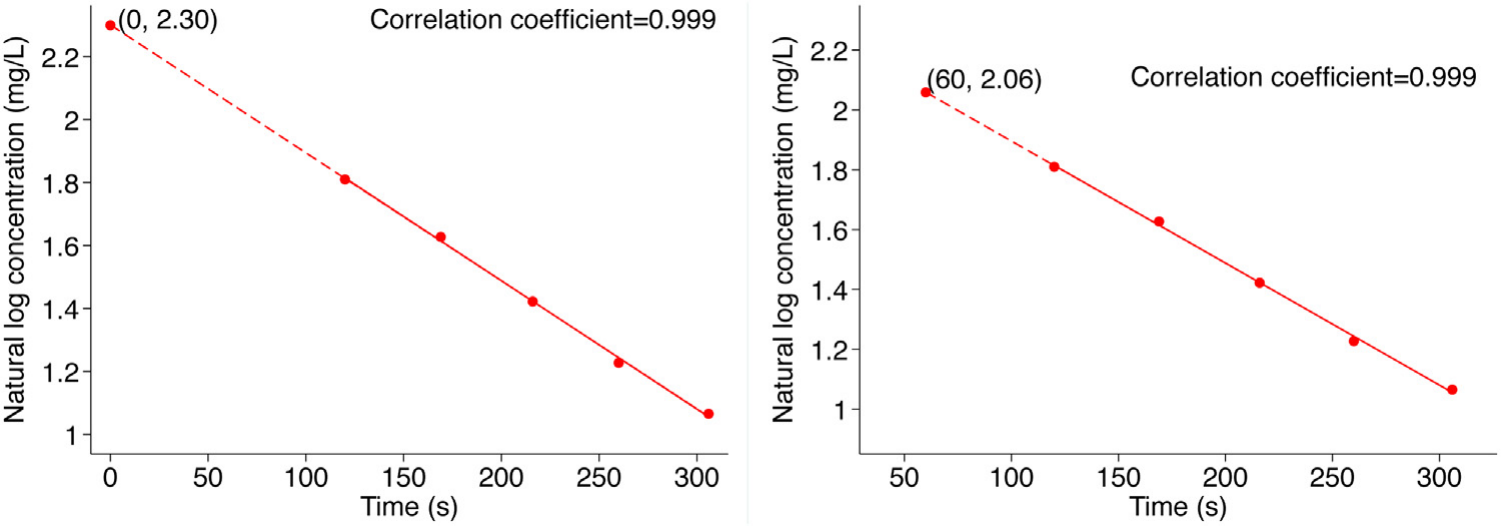
The decay curve for plasma ICG concentrations back-extrapolated to time, t = 0 (left) and t = 1 min (right) using data collected from t = 2–5 minutes (example from one participant).

We constructed a standard curve of absorbance against standard concentrations (Fig. 2) and used it to estimate the concentrations of serially collected plasma samples, obtained from t = 2–5 min, for each participant. The concentrations of ICG in serial plasma samples were transformed into natural logs and plotted against the time they were collected, from t = 2–5 min post-injection (converted to seconds, exact time of collection used). Traditionally, plasma volume estimation with ICG is made by back-extrapolation to time t = 0 min [14,16,24,39,41]. However, this method has been shown in some studies to underestimate PV partly due to incomplete ICG mixing at this time (i.e., the time of injection) [13,42]. To overcome this problem, other researchers resorted to using a tourniquet (prior to ICG injection) to create a state of reactive hyperemia to speed up intravascular mixing and distribution of ICG [14,16]. Another solution, shown by Polidori and Rowley, is to use backward-extrapolation to time t = 1 min, which produces consistent and more accurate PV than back-extrapolating to t = 0 [23]. In this paper, we back-extrapolated the ICG concentration to both t = 0 and t = 1 min so that the data is comparable to either approach for future work. We also showed back-extrapolation graphs for both timepoints (Fig. 3). The PV (L) for the participant was thus calculated as:

PV = D/C_0_

(where D = Dose of ICG administered (mg) and C_0_ = Plasma concentration of ICG (mg/L) at t_0_ = 0 min, back-transformed from natural log). This procedure was repeated for each participant with a new calibration curve constructed from the participant’s plasma (Suppl. 2). PV was also calculated by weight and body surface area [43]. We also examined the relationship between PV and BSA, because strong association between the two have been reported [44,45].

Overall, ˜1.5 mL of pre-injection plasma (blank) and 0.2 mL of each post-injection plasma sample was sufficient to measure PV. This included five standard concentrations (2.5 mg/L–10 mg/L), which were sufficient for the estimation of PV for all subjects. The total of ˜2.5 mL of plasma needed per participant was much lower than the amount used in other studies [16,40]. While this is a small amount of plasma, we recommend a total of 14 mL (4 mL pre-injection and 2 mL × 5 post-injection) of blood collection to allow for repeat testing if needed. As well, although we used 5 post-injection samples, we found that 3 samples would be sufficient (i.e., if we missed a blood draw during the procedure). The laboratory work for each participant measurement of PV took approximately two hours to complete, after blood collection.

## Results

### Characteristics of the study sample

A total of nine women enrolled and were included in the analyses. Eight women self-identified as white and one as African-American. Participants were college educated (n = 5) or were undergraduate students (n = 4). Two women were married (others were never married); all women were nulliparous except one. The mean ± SD age of participants was 25.0 ± 4.5 years, BMI was 23.5 ± 2.9 kg/m^2^, and total body fat was 28.6 ± 5.0%. At the beginning of the visit, mean systolic blood pressure was 106 ± 8 mmHg and mean diastolic blood pressure was 71 ± 4 mmHg. Table 3 presents the PV of each participant sorted from low to high.

**Table 3:** Participants and plasma volume (n = 9)^a^.

Age (years)	Weight (kg)	BMI (kg/m^2^)	Body fat (%)	SBP (mmHg)	DBP (mmHg)	Plasma volume (mL)^b^	Plasma volume (mL)^c^
26	54.7	21.6	25.6	109	70	1250	1500
28	53.5	20.3	21.5	99	69	1220	1570
21	63.6	24.2	29.4	98	66	1200	1600
21	65.5	26.1	27.8	123	75	1510	1940
18	63.6	22.1	26.8	103	66	1600	2000
23	65.0	22.5	31.4	109	79	1700	2200
27	64.9	23.8	26.1	109	75	1640	2350
31	79.5	29.6	39.7	108	74	2000	2660
30	63.6	21.4	29.4	101	69	2410	2780
**25.0 ± 4.5**	**63.8 ±** **7.4**	**23.5 ± 2.9**	**28.6 ±** **5.0**	**106 ± 8**	**71 ±** **4**	**1614 ±** **397**	**2067 ± 470**

a Plasma volume values are sorted from lowest to highest; Mean ± SD (reported in bold in the last row for each variable); BMI, body mass index; SBP, systolic blood pressure; DBP, diastolic blood pressure.

b Plasma volume extrapolated t = 0.

c Plasma volume extrapolated t = 1 min (60 s).

**Fig. 4:**
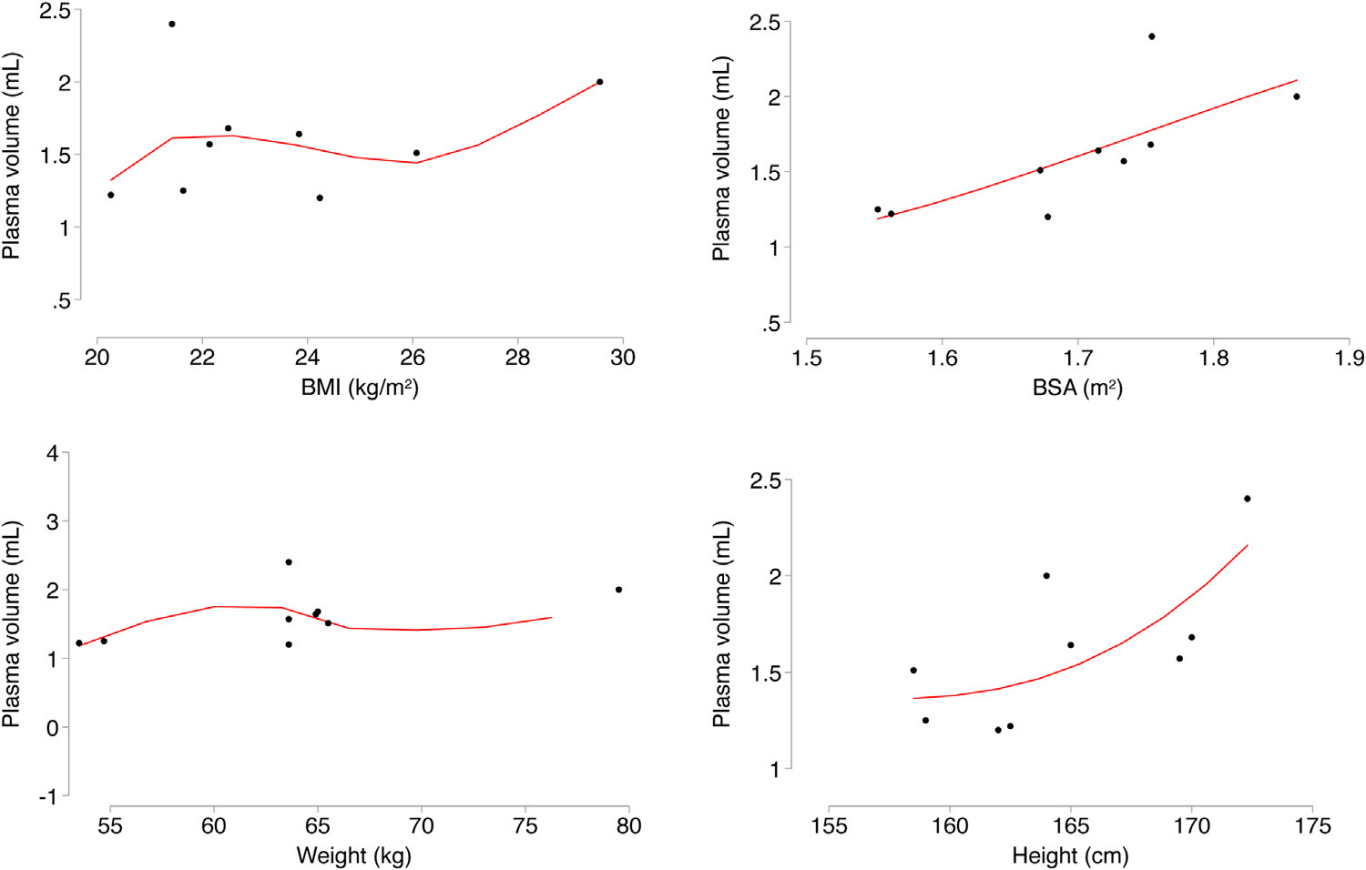
Local polynomial regression plot (regression line) of height, weight, BMI and BSA against plasma volume (at time, t = 0). The Spearman’s correlation coefficient and regression estimates were: BMI on plasma volume (r = 0.23, *P* = 0.547; ß = 0.032, 95% CI: -0.088, 0.152); BSA on plasma volume (r = 0.74, *P* = 0.022; ß = 3.023, 95% CI: 0.597, 5.457); weight on plasma volume (r = 0.57, *P* = 0.111; ß = 0.030, 95% CI: −0.009, 0.069), and height on plasma volume (r = 0.68, *P* = 0.043; ß = 0.055, 95% CI: 0.002, 0.107). Body mass index (kg/m^2^); BSA, body surface area (m^2^).

The mean ± SD of the correlation coefficient for the standard curve was 0.989 ± 0.023; 6 out of 9 were >0.99. The correlation coefficient for the decay curve was 0.991 ± 0.013; 7 out of 9 were >0.99. The mean ICG elimination rate constant (k) was 0.25 ± 0.06 /min and the (hepatic) clearance of ICG was 402 ± 119 mL/min (t = 0). The mean coefficient of variation for PV across the nine participants was 1.7%. The mean PV for t = 0 was 1608 ± 394 mL (or 2067 ± 470 mL, when extrapolated to time, t = 1 min). PV by body size was 25 ± 5 mL/kg body weight and 941 ± 193 mL/m^2^ body surface area (t = 0). The relationships between PV and anthropometric measures are presented in Fig. 4. The correlation was particularly strong for BSA and plasma volume (r = 0.74, *P* = 0.022). The correlation coefficients were high for both the calibration curves and ICG decay curves (back-extrapolation). A sample standard curve and ICG decay curve from the study are shown in Fig. 2, Fig. 3, respectively. The dye had a circulatory half-life of 2.9 ± 0.9 min and the ICG-plasma disappearance rate was 25.4% per minute. No participant reported any adverse events for the duration of the study.

## Discussion and conclusion

This study developed and field tested an efficient method for measuring PV using ICG dye among healthy, non-pregnant women. We have shown that the measurement of PV can take less than 2 h to accomplish compared to commonly used methods that can take several hours to obtain results [[46], [47], [48]]. This makes the method practical for research, however challenges for use in clinical settings remain. Some have used the non-invasive pulse dye densitometry [31,32,[49], [50], [51], [52], [53], [54]] to avoid the need for blood draws when using ICG, which deserves further consideration for application to PV estimation. This densitometry method has not yet been approved by the FDA for use in the US. Similarly, BVA-100 (Blood Volume Analyzer), a semi-automated system for blood volume analysis reduces the time required to measure blood volume using ^131^I-labeled HSA as tracer. This method has been approved by the FDA [12,55] but because it uses a radioactive iodine isotope, there are concerns using it in some populations like pregnant women and young children. We avoided using radioactive isotopes because our long-term goal is to have a method that works for maternal and child health research. Another concern in clinical settings is overestimation of PV due to the escape of albumin-bound ICG into the interstitial space. This has been documented in patients with capillary leaks (e.g., sepsis, burns, trauma, and inflammation) [51,56]. In healthy patients, losses are minimal and do not appear to affect PV estimates [24,56,57].

By adjusting methods and concentrations, we found that a small quantity of plasma (˜2.5 mL) can be used to measure PV – needing only 14 mL of blood to be drawn from each person. Further, our method reduced the number of post ICG-injection samples needed to 5, which is fewer than other methods that require 7–10 post-injection samples [16,24,40,[58], [59], [60]]. PV can be estimated with as few as 3 post-injection plasma samples, but having 5 samples improves the accuracy of estimates. Though we collected blood every 45 s, other time intervals could be used. The most important factor is that timing of blood collection should be precisely recorded. Altogether, we have reduced the total amount of blood needed, which is helpful in all populations but can be important in certain clinical cases.

Currently, no universally accepted standard values for comparing PV across age and gender exist. However, our measured values (mean 2067 mL using the t = 1 extrapolation) are comparable to estimates from Pearson et al. who estimated PV among more than 400 males and females from different equations (for 67 females with PV data, the estimates range from 2029 mL to 2744 mL) [61]. Although we did not compare our results with the method recommended by ICSH or other common methods for measuring PV, our estimates are comparable to what is commonly reported in literature for the age group examined. Furthermore, previous studies have shown that PV estimates from ICG were comparable to the ICSH recommended method – ^125^I-HSA [14] and/or that of Evans blue dye [13] and our goal was not to re-validate the ICG measurement.

Taken together, the short circulatory half-life and high plasma disappearance rate give further support of ICG rapid clearance from the plasma, and safety when used in humans. This also makes it an ideal tracer for repeated use in plasma volume determinations. The half-life reported in this study was consistent with previous estimates [16,17]. ^125^I–HSA, has a circulatory half-life of 60 days [11]. This makes repeated use of the method unacceptable because of the possibility of accumulation in the body. Over the years, interest in and use of ICG has been increasing particularly in clinical settings. A review in 2012 showed that in 1970 there were 38 studies on the use of ICG in PubMed, compared to 397 studies in 2010 [62]. As more evidence becomes available in PV measurements, ICG use will become more common.

In conclusion, ICG is a safe, efficient method for the measurement of PV in adults. As reported here, we successfully piloted the method in nine participants. Since then, we have used the same method in a longitudinal study of 35 women with 3 visits across the menstrual cycle, resulting in over 100 PV measurements. In our method development, we have further improved the method by reducing the amount of time and the volume of plasma needed to measure PV. PV can be estimated within 2 h using only 5 post-injection blood draws and only ˜2.5 mL of plasma per participant. ICG should be a recommended method for PV measurement in future research.

## Funding

The Pennsylvania State University, College of Health and Human Development.

## Conflicts of interest

Authors declared no conflict of interest exist.

## Ethical considerations

The protocol was approved by The Office for Research Protections (ORP) at The Pennsylvania State University (STUDY00004051) and conducted in line with the Declaration of Helsinki. All participants provided written informed consent before enrolling into the study.
