# Supplementary material for: An efficient method for measuring plasma volume using indocyanine green dye
Source: MethodsX. 2019 May 8;6:1072–83. doi: 10.1016/j.mex.2019.05.003 (PMC6526294; doi:10.1016/j.mex.2019.05.003)
Supplement: Supplementary file 1 [file mmc1.docx]

**Supplementary material**

Supplement 1: Spreadsheet template for PV calculations

Supplement 2: Video of the blood collection, ICG injection, 3-way stopcock system replacement, and post-ICG injection blood collection.
